# Accuracy of parents in measuring body temperature with a tympanic thermometer

**DOI:** 10.1186/1471-2296-6-3

**Published:** 2005-01-11

**Authors:** Joan L Robinson, Hsing Jou, Donald W Spady

**Affiliations:** 1 Department of Pediatrics and Stollery Children's Hospital, 2C3 Walter MacKenzie Centre, 8440-112 St., Edmonton, Alberta, T6G 2B7 Canada

## Abstract

**Background:**

It is now common for parents to measure tympanic temperatures in children. The objective of this study was to assess the diagnostic accuracy of these measurements.

**Methods:**

Parents and then nurses measured the temperature of 60 children with a tympanic thermometer designed for home use (home thermometer). The reference standard was a temperature measured by a nurse with a model of tympanic thermometer commonly used in hospitals (hospital thermometer). A difference of ≥ 0.5 °C was considered clinically significant. A fever was defined as a temperature ≥ 38.5 °C.

**Results:**

The mean absolute difference between the readings done by the parent and the nurse with the home thermometer was 0.44 ± 0.61 °C, and 33% of the readings differed by ≥ 0.5 °C. The mean absolute difference between the readings done by the parent with the home thermometer and the nurse with the hospital thermometer was 0.51 ± 0.63 °C, and 72 % of the readings differed by ≥ 0.5 °C. Using the home thermometer, parents detected fever with a sensitivity of 76% (95% CI 50–93%), a specificity of 95% (95% CI 84–99%), a positive predictive value of 87% (95% CI 60–98%), and a negative predictive value of 91% (95% CI 79–98 %). In comparing the readings the nurse obtained from the two different tympanic thermometers, the mean absolute difference was 0.24 ± 0.22 °C. Nurses detected fever with a sensitivity of 94% (95 % CI 71–100 %), a specificity of 88% (95% CI 75–96 %), a positive predictive value of 76% (95% CI 53–92%), and a negative predictive value of 97% (95%CI 87–100 %) using the home thermometer. The intraclass correlation coefficient for the three sets of readings was 0.80, and the consistency of readings was not affected by the body temperature.

**Conclusions:**

The readings done by parents with a tympanic thermometer designed for home use differed a clinically significant amount from the reference standard (readings done by nurses with a model of tympanic thermometer commonly used in hospitals) the majority of the time, and parents failed to detect fever about one-quarter of the time. Tympanic readings reported by parents should be interpreted with great caution.

## Background

Parents and health care workers use temperatures measured by parents at home to determine if a child requires medical assessment. Health care workers sometimes make decisions about the need for investigations and hospital admission based on the temperature that parents report. This is especially true in the newborn or in the immunocompromised patient, where any temperature above the normal range can be indicative of bacterial infection.

Health care workers have used commercial tympanic thermometers in hospitals for over 15 years. These thermometers are quick and easy to use and minimize the risk of nosocomial infection. Tympanic thermometers are now available for home use at a cost of about $50 US. There are no published studies comparing measurements obtained with tympanic thermometers designed for home use to measurements obtained with other types of thermometers, or comparing readings obtained by parents with these instruments to those obtained by health care workers. The primary objective of this study was to determine the diagnostic accuracy of readings obtained by parents with a tympanic thermometer designed for home use.

## Methods

The Health Research Ethics Board of the University of Alberta approved this study and parents and older children signed a consent form.

### Reference standard

Traditionally, rectal temperatures have been used as the reference standard in pediatrics. However, readings done by a nurse using a model of tympanic thermometer commonly used in hospitals were chosen as the reference standard for this study as they are easier to obtain than are rectal temperatures and tend to be closer to PA temperatures when the patient is febrile [1] or when the temperature is changing [2,3], although the opposite may be true in the steady state [4].

### Subjects

Parents of patients from the General Pediatric Clinic, the Emergency Department, or on the inpatient units of the Stollery Children's Hospital were eligible for the study. Parents of febrile or afebrile children aged 6 months to 16 years were approached on the days when the study nurse was available, but preference was given to patients who were being assessed because of fever. Parents were not approached if it would be inconvenient for them to participate in the study, or if they did not communicate in English, but there were no other exclusion criteria.

### Study procedures

After informed consent was obtained from the parent (and the child when practical), the parent was given the instruction sheet from the Braun Thermoscan (Thermoscan Inc., San Diego, CA) tympanic thermometer (home thermometer) to read. They were then asked to measure their child's temperature when they felt they understood the instructions. They then recorded the temperatures they measured on a sheet of paper. Without looking at this sheet of paper, one of three trained research nurses then measured the child's temperature using the same thermometer. The nurse then also measured the child's temperature with CORECHECK (ALARIS Inc., San Diego, CA) tympanic thermometer (hospital thermometer). All readings were done in the same ear with a new probe cover.

### Outcome measures

The mean absolute difference between the parents' reading and the reading by the nurse using the same thermometer was calculated. Readings were then analyzed separately for the febrile children. A temperature of ≥ 38.5 °C on the reading taken by the nurse using the hospital thermometer was defined as a fever. The percentage of times that the readings differed by 0.5 °C or more was calculated as this was considered a clinically significant difference.

Even if the parent and the nurse obtained similar readings from the home thermometer, it would still be possible that these readings are inaccurate, as there are no published studies demonstrating the accuracy of this thermometer. Therefore, the readings obtained by the nurse and the parent from the home thermometer were compared to those obtained by the nurse using the hospital thermometer (the reference standard for the study). If the readings obtained by the parent differed considerably from those obtained by the nurse, but the nurse then obtained very consistent readings with the two different thermometers, one could conclude that the parents were inaccurate because of human error. On the other hand, if the nurse obtained inconsistent readings with the two different thermometers, this would suggest that either instrument or human error could be the problem.

The measures of central tendency were determined, and the intraclass correlation coefficient determined for the three sets of readings. The three sets of measurements were also compared using the Bland-Altman method [5] where the differences between two measurements are plotted against the average of the measurements.

### Sample size calculation

It was not possible to calculate a sample size, as the expected standard deviation between the three sets of readings could not be predicted. We estimated that a sample size of 60 children would be adequate.

## Results

### Characteristics of study subjects

Sixty parents were approached and all agreed to participate in the study. Their children were 35 males and 25 females aged 5 months to 15 years (median 3 years). Although the majority of patients were being assessed because of fever, only 17 children were febrile at the time of the study. All 3 measurements were obtained within 5 minutes for 55 of the 60 cases.

### Main results

**Figure 1:**
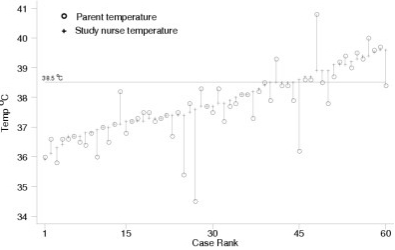
Comparison of readings done by a parent to readings done by a nurse using a home tympanic thermometer

Table 1 shows the results of the study. The mean absolute difference between the temperature recorded by the parent and the temperature recorded by the nurse using the home thermometer was 0.44 ± 0.61 °C (Figure 1). The difference was similar in febrile and afebrile children. The higher reading was from the parent in 20 of the 60 cases. Parents detected fever with a sensitivity of 76% (95% CI 50–93%), a specificity of 95% (95% CI 84–99%), a positive predictive value of 87% (95% CI 60–98%), and a negative predictive value of 91% (95% CI 79–98 %) (Table 2).

**Table 1:** Comparison of results of temperatures measured by parents using a tympanic thermometer designed for home use, a nurse using the same thermometer, and the same nurse using a tympanic thermometer designed for hospital use

	Mean absolute difference (°C)	Range of absolute differences (°C)	% of time where absolute difference ≥ 0.5 °C
Parent versus nurse using home tympanic thermometer All children n = 60	0.44 ± 0.61	0.0 – 3.1	33%
Parent versus nurse using home tympanic thermometer Afebrile children n = 43	0.44 ± 0.65	0.0–3.1	35%
Parent versus nurse using home tympanic thermometer Febrile children n = 17	0.45 ± 0.51	0.10 – 1.9	29%
Nurse using home thermometer versus same nurse using hospital thermometer n = 60	0.24 ± 0.22	0.0 – 1.0	13%
Parent using home thermometer versus nurse using hospital thermometer N = 60	0.51± 0.63	0.0 – 3.4	72%

The mean absolute difference between the temperatures measured by the nurse using the home thermometer versus the hospital thermometer was 0.24 ± 0.22 °C. The higher reading was from the hospital thermometer in 25 of the 60 cases. There was agreement between the readings taken by the nurse from the two thermometers with regard to the presence of fever, except in one case where one reading was 38.4 °C and the other was 38.6 °C (Table 2). Nurses detected fever with a sensitivity of 94 % (95 % CI 71–100 %), a specificity of 88 % (95% CI 75–96 %), a positive predictive value of 76% (95% CI 53–92 %), and a negative predictive value of 97% (95% CI 87–100 %) (Table 2). In comparing the readings obtained by the parent using the home thermometer to those obtained by the nurse using the hospital thermometer, the mean absolute difference was 0.51 ± 0.63 °C, with 72% of the readings differing by ≥ 0.5 °C.

**Table 2:** Ability of parents and nurses to detect fever using a home tympanic thermometer, with a hospital tympanic thermometer being the reference standard

	Nurse reported fever on hospital thermometer	Nurse reported no fever on hospital thermometer
Parent reported fever on home thermometer	13	2
Parent reported no fever on home thermometer	4*	41
Nurse reported fever on home thermometer	16	5
Nurse reported no fever on home thermometer	1**	38

*Readings by parents were 37.8 °C, 37.9 °C, and 38.4 °C twice with corresponding hospital thermometer readings being 38.6 °C, 38.5 °C, 38.6 °C, and 39.6 °C

**Reading was 38.4 °C on home thermometer and 38.6 °C on hospital thermometer

**Figure 2:**
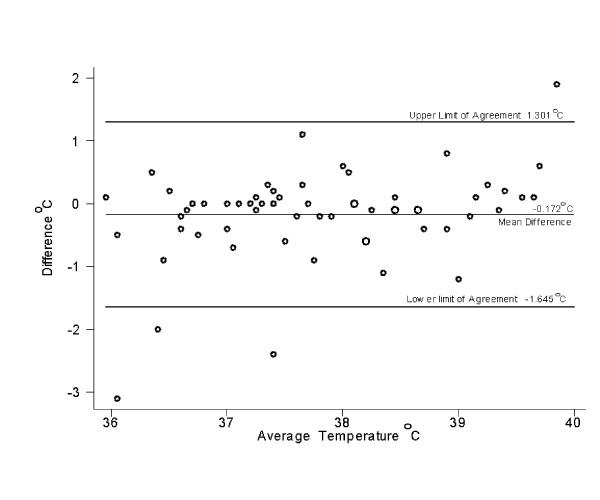
Scatter plot of the difference between temperature measured by a parent and that measured by a nurse using a thermometer designed for home use

**Figure 3:**
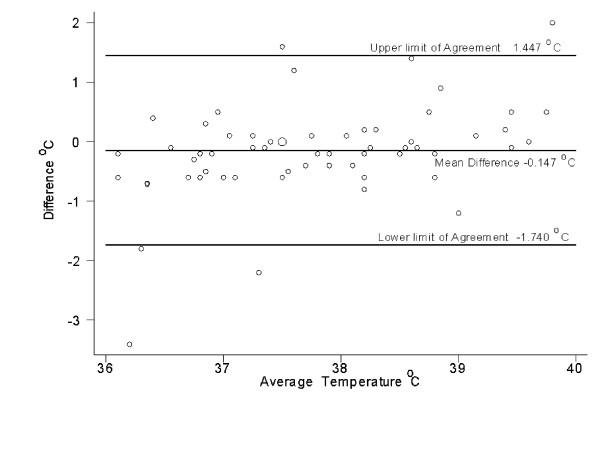
Scatter plot of the difference between temperature measured by a parent using a thermometer designed for home use and a nurse using a thermometer designed for hospital use

**Figure 4:**
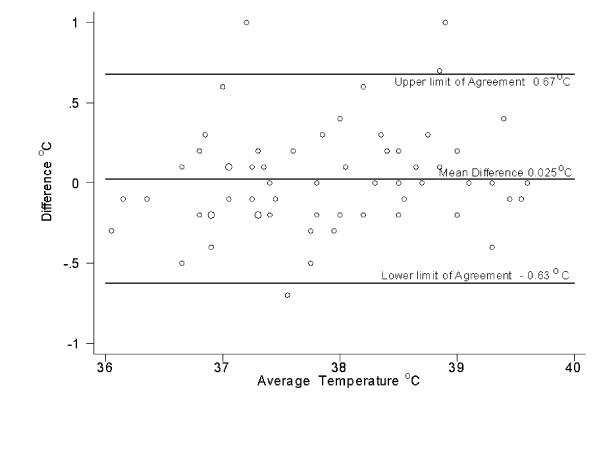
Scatter plot of the difference between temperature measured by a nurse using a thermometer designed for home use and a a thermometer designed for hospital use

The intraclass correlation coefficient for the three sets of readings was 0.80. Figures 2, 3, and 4 show that the variation between the readings was consistent at different temperatures.

## Discussion

It is now common for parents to report a child's body temperature as measured by a tympanic thermometer designed for home use. The only published study looking at the reliability of parents at measuring tympanic temperatures reported on the consistency of readings, rather than comparing the temperatures measured by parents to those measured by health care workers or by another method [6].

The current study showed that readings obtained by parents differ from those obtained by a nurse using the same instrument by a mean of 0.44°C, with 33% of the readings differing by a clinically significant amount (0.5°C or more). The absolute differences were similar for febrile and afebrile children. Using the reference standard of a tympanic temperature measured by a nurse with a model of thermometer commonly used in hospitals, the parents did not detect a fever in four of 13 cases (although in two of these cases, the reading by the parent was just 0.1 °C below our definition of fever). This suggests that the readings obtained by a parent with a tympanic thermometer are sometimes not reliable. In fact, previous studies showed that parents' subjective assessment of whether their child had a fever had a sensitivity of 81.8% [7] and 88.9% [8], which is similar to the 76 % sensitivity in the current study when parents used a tympanic thermometer.

In comparing the readings taken by a nurse with a home tympanic thermometer to a hospital tympanic thermometer, the mean absolute difference was 0.24 °C, with 13 % of the readings differing by a clinically significant amount. It is not clear if this degree of discrepancy is to be expected when a skilled operator takes two tympanic readings, or if it was a true difference between the two types of tympanic thermometer. The nurse would have identified all but one of the 17 children with fever as being febrile with either thermometer, and that child had a temperature of 38.4 °C on one thermometer and 38.6 °C on the other thermometer. Therefore, it appears that with a skilled operator, the tympanic thermometer designed for home use is reasonably reliable, and is likely to detect fever even if the actual reading is not always accurate.

The chief limitation of this study is that readings obtained by a nurse from the hospital tympanic thermometer must be reliable for the results of this study to be accurate. Some studies have concluded that tympanic thermometers do not give an accurate measurement of body temperature [7,9,10]. However, the reference standard in these studies (axillary or rectal temperature) may not be ideal. Studies have shown that when used by a skilled operator on a cooperative patient with rapidly changing body temperature, the readings from models of tympanic thermometers commonly used in hospitals more closely approximate pulmonary artery or esophageal temperature than do rectal or axillary temperatures [2,3]. Therefore, a tympanic temperature measured with a hospital tympanic thermometer was considered the best available reference standard for this study. However, if the nurses used incorrect technique, this would affect the results of the study. Falsely high readings with a properly calibrated tympanic thermometer should only occur if the thermometer itself has been stored above room temperature, so the fact that in 25 of the 60 cases the parent obtained a higher reading than did the nurse suggests the possibility that the nurses were not using ideal technique. Another limitation is that we could not blind the nurses for comparing their own two temperature readings. There is some evidence that falsely low readings can be obtained if tympanic readings are obtained in rapid succession as placement of the thermometer results in local cooling, so it is possible that readings would have been more comparable had we waited at least 2 minutes between readings [11]

It is possible that parents would have performed better had they leisurely read the instructions in their own home rather than doing so in a hospital-based setting, or had they had time to practice. However, it is also possible that they performed better than they would have in the home, as they knew evaluation of their performance was occurring. It is also possible that parents who would choose to buy a tympanic thermometer will differ from our study population. It is not clear what advice to give to parents about purchasing a thermometer. The ability of parents from the inner city to correctly use and read a mercury glass thermometer was poor in previous studies [12,13]. An electronic thermometer is easier to read and is now relatively inexpensive, but may not be as accurate as a mercury thermometer [14]. The results of the current study suggest that readings obtained by parents with a tympanic thermometer often differ by a clinically significant amount from readings obtained by nurse. Despite their ease of use, there are reports of parents inserting tympanic thermometers in the rectum [15]. It is not clear if parents could more accurately use an infrared temporal artery thermometer. A study showed that using the highest of three temporal artery temperature obtained by a parent or nurse, a reading of ≥ 37.8 °C was 97% sensitive and 84% specific for detecting a rectal temperature of ≥ 38.5 °C, and that the limits of agreement for temperatures obtained by parents versus nurses using this thermometer were -0.6 °C to 0.7 °C [16]. This is lower than the limits of agreement for the current study, but it is possible that the parents would have performed better had we recorded the highest of three readings.

## Conclusions

With the recent outbreak of Severe Acute Respiratory Syndrome, rapid measurement of body temperature (sometimes by personnel who are not health care workers) has become an important tool to indicate if people can board airplanes and if staff can work. It is therefore important to determine the accuracy of non-health care workers at measuring body temperature. This study showed that the temperatures measured by parents with a tympanic thermometer often differ by a clinically significant amount from those measured by a nurse with a tympanic thermometer. There is not a consistent gradient or direction of the gradient between the two temperatures, which makes it difficult to interpret the temperatures reported by parents. There was a smaller gradient between the readings taken by a nurse with a tympanic thermometer designed for home use compared with those taken with a model of tympanic thermometer commonly used in hospitals, but there were still potentially clinically significant discrepancies 14% of the time. This makes it difficult to conclude if the poor performance of the parents relates entirely to human error, or if there is also an element of instrument error. The fact that in comparison with our reference standard (tympanic temperature measured by the nurse with a hospital thermometer) the readings taken with the home thermometer by the nurse correlated much better than those taken by the parent suggests that the parents did not use ideal technique. In any case, one should interpret temperatures taken by parents with a tympanic thermometer with great caution. Although parents will detect the majority of fevers with these instruments, the absolute numbers obtained may not be accurate. Again, we find that in pediatrics, the clinical picture is a more useful piece of information than are "the numbers"!

## Abbreviations

°C – degrees Celsius

PA: pulmonary artery

## Competing interests

The author(s) declare that they have no competing interests.

## Authors' contributions

JLR wrote the protocol and the manuscript. HJ helped with patient recruitment and reviewed the manuscript. DWS did the statistical analysis and reviewed the manuscript.

## Pre-publication history

The pre-publication history for this paper can be accessed here:


